# Hyperhalophilic Diatom Extract Protects against Lead-Induced Oxidative Stress in Rats and Human HepG2 and HEK293 Cells

**DOI:** 10.3390/ph16060875

**Published:** 2023-06-13

**Authors:** Wassim Guermazi, Saoussan Boukhris, Neila Annabi-Trabelsi, Tarek Rebai, Alya Sellami-Kamoun, Waleed Aldahmash, Gabriel Ionut Plavan, Abdel Halim Harrath, Habib Ayadi

**Affiliations:** 1 Laboratory of Marine Biodiversity and Environment, Department of Life Sciences, Faculty of Sciences, University of Sfax, Street of Soukra Km 3.5, Sfax CP 3000, Tunisia; 2 Laboratory of Enzyme Engineering and Microbiology, Department of Life Sciences, National Engineering School of Sfax, University of Sfax, Sfax CP 3038, Tunisia; 3 Laboratory of Histology-Embryology, Faculty of Medicine, Magida Boulila Street, Sfax CP 3028, Tunisia; 4 Department of Zoology, College of Science, King Saud University, Riyadh 11451, Saudi Arabia; 5 Faculty of Biology, Alexandru Ioan Cuza University, 700505 Iasi, Romania

**Keywords:** microalgal extract, human cell line, fatty acid, lead exposure, oxidative stress, rats

## Abstract

This work investigated the protective effects of microalga *Halamphora* sp. extract (HExt), a nutraceutical and pharmacological natural product, on human lead-intoxicated liver and kidney cells in vitro and in vivo in Wistar rats. The human hepatocellular carcinoma cell line HepG2 and the human embryonic kidney cell line HEK293 were used for the in vitro study. The analysis of the fatty acid methyl esters in the extract was performed via GC/MS. The cells were pretreated with HExt at 100 µg mL^−1^, followed by treatment with different concentrations of lead acetate, ranging from 25 to 200 µM for 24 h. The cultures were incubated (5% CO, 37 °C) for 24 h. Four groups, each containing six rats, were used for the in vivo experiment. The rats were exposed to subchronic treatment with a low dose of lead acetate (5 mg kg^−1^ b.w. per day). Pretreating HepG2 and HEK293 cells with the extract (100 µg mL^−1^) significantly (*p* < 0.05) protected against the cytotoxicity induced by lead exposure. For the in vivo experiment, the biochemical parameters in serum—namely, the level of malondialdehyde (MDA), and the activities of superoxide dismutase (SOD), catalase (CAT), and glutathione peroxidase (GPx)—were measured in the organ homogenate supernatants. HExt was found to be rich in fatty acids, mainly palmitic and palmitoleic acids (29.464% and 42.066%, respectively). In both the in vitro and in vivo experiments, cotreatment with HExt protected the liver and kidney cell structures and significantly preserved the normal antioxidant and biochemical parameters in rats. This study discovered the possible protective effect of HExt, which could be beneficial for Pb-intoxicated cells.

## 1. Introduction

The brown microalga *Halamphora* sp., Bacillariophyceae class (diatoms), when cultivated in high-salinity medium (100 p.s.u.), has been proven to contain moderate percentages of proteins, lipids, sugars, and minerals, as well as significant levels of polyphenols, flavonoids, chlorophyll, carotenoids, and bioactive fatty acids such as eicosapentaenoic acid [1]. Due to extreme salinity conditions and a phototrophic mode of growth, halophilic microalgae are exposed to free radical and high oxidative stresses [2]. This has evolved the halophilic diatom in developing a natural protective system such as the accumulation of carotenoids, mainly fucoxanthin [3,4]. This component is useful for human supplementation as it is not synthesized internally by individuals [5]. The antioxidant, anticancer, anti-inflammatory, and anti-obesity activities of fucoxanthin from micro–macroalgae and medicinal plants have been largely documented [6,7,8,9]. The fatty acid compositions of diatom lipids, which are comparable to those found in plant oils and other microalgae, have recently attracted attention as a new source of bioactive chemicals for use in nutraceutical and pharmaceutical applications [10,11,12].

The prominent contents of bioactive compounds in this microalga favor its possible valorization in food additives and pharmaceutical products [1]. *Halamphora* sp. is used as a feed supplement in the aquaculture of the *Penaeus monodon* postlarval hatchery system [13]. In addition, the consumption of fatty acids as a dietary supplement or food ingredient has the potential to provide health benefits, including a reduction in pain related to inflammation [14].

On the other hand, attention has also been drawn to the protective effects of natural antioxidants against chemically induced oxidative damage, especially when the generation of free radicals is involved. Over the last 50 years, the ongoing inflow of xenobiotics into the environment has resulted in an up to eight times increase in the number of recorded liver diseases [15].

In fact, humans and animals are exposed daily to oxidants such as water, air pollutants, and heavy metals, including lead (Pb), which is one of four metals that have the most damaging effects on human health [16]. Lead is a poison of polytropic behavior and is capable of accumulation in an organism, thus causing several negative effects, such as lesions in the hematopoietic, nervous, digestive, excretory, and other systems [17]. Nosologies related to liver dysfunction rank as some of the leading positions among occupational diseases [18].

Both occupational and environmental exposures to heavy metals remain as serious problems for many developing and industrializing countries [19]. Similar to other heavy metals, lead cannot be degraded or destroyed, and it is present everywhere in the environment, including in water, soil, and in products manufactured with lead [20]. In this paper, this environmental contaminant was chosen for cell exposure in order to induce the production of reactive oxygen species (ROS). Several studies have shown that, in animals and humans, lead alters the activities of antioxidant enzymes, namely SOD, CAT, and GPx, and antioxidant molecules such as GSH [21]. In addition, awareness of the harmful effects of lead on the health of animals and humans is increasing as lead exposure has many undesirable effects, including hepatic and renal effects [22].

Recently, certain experiments conducted in animals have proven that cyanonabacteria, such as Spirulina (*Arthrospira*), can protect against lead intoxication [23,24] and the effects of other heavy metals [25] and toxic chemicals [26]. Belhaj et al. [27] and Gammoudi et al. [28] reported that the polysaccharides and phycocyanin extracted from halophilic cyanobacteria *Phormidium versicolor* may induce nephro-hepatoprotective actions after the intoxication of rats and cell lines (HepG2) by cadmium. However, in vivo studies evaluating the extract of diatoms are scarce.

In this respect, this research aims to investigate *Halamphora* sp. extract in terms of its role in protecting against lead intoxication, its phytochemical and fatty acid composition, and its effects on the biochemical and histological damage caused by lead acetate rats and human liver and kidney cells.

## 2. Results

### 2.1. Biochemical Composition of Diatom Halamphora Extract

The total phenol, flavonoid, pigment, and fatty acid contents in *Halamphora* sp. extracts are summarized in Table 1. The ethanolic extract of this Bacillariophyceae showed the highest phenolic and flavonoid contents—38.27 ± 2.21 mg GAE g^−1^ extract and 17.69 ± 0.70 mg QE g^−1^ extract, respectively. The chlorophyll (a) content in this extract almost reached the 6.11 mg g^−1^ extract, and the carotenoid content was prominent at approximately 2.19 mg g^−1^ extract. The fatty acid profile of HExt is composed of saturated fatty acids, monounsaturated fatty acids, and polyunsaturated fatty acids (Table 1). The palmitic (C16:0) and palmitoleic acids (C16:1) accounted for more than 71% of the total fatty acids. The saturated fatty acids accounted for 38.677%, with a predominance of palmitic acid C16:0 (29.464%). The HExt was found to be rich in monounsaturated fatty acids (47.893%), with a predominance of palmitoleic acid C16:1 (42.066%), and the extract also contained a significant percentage of polyunsaturated fatty acids (10.960%). The HExt exhibited a high level of ecosapentaenoic acid 20:5(n-3) (6.07%), followed by arachidonic acid (2.87%) (Table 1).

### 2.2. Cytotoxicity Assay

**Figure 1:**
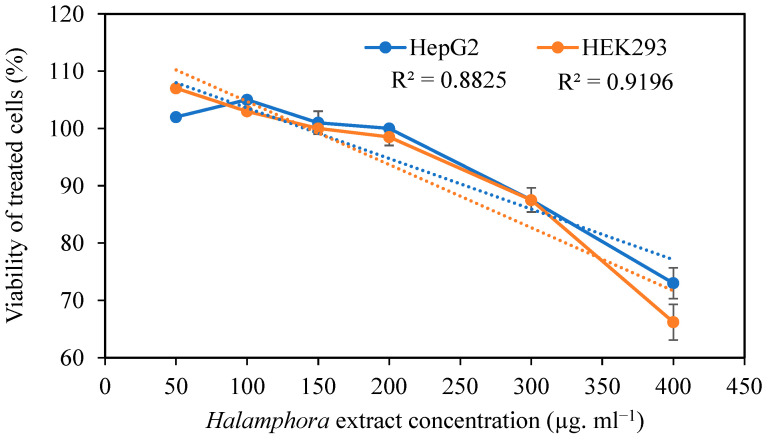
Effect of *Halamphora* extract on the viability (%) of HepG2 and HEK293 cells. Data are expressed as the mean ± SD. R^2^: coefficient of determination. Dashed line corresponds to the curve of regression.

The effects of *Halamphora* extract on HepG2 and HEK293 cell viability are shown in Figure 1. In the present study, the viability of HepG2 and HEK293 cells was not significantly inhibited, up to 200 µg mL^−1^, by HExt. Six concentrations of extract—namely 50, 100, 150, 200, 300, and 400 µg mL^−1^—were used to test their effects on the viability of both cell lines. The results revealed that extract concentrations of <200 µg mL^−1^ did not show any significant (*p* < 0.05) effect on cell viability when compared to the control cell culture. The use of 50 and 100 µg mL^−1^ of HExt exhibited the highest level of viability for HEK293 and HepG2 cells, respectively (Figure 1). At the highest tested concentration of this extract (400 µg mL^−1^), the viabilities of HepG2 and HEK293 cells were 73% and 67%, respectively.

### 2.3. Effect of Lead Acetate on Cell Viability

**Figure 2:**
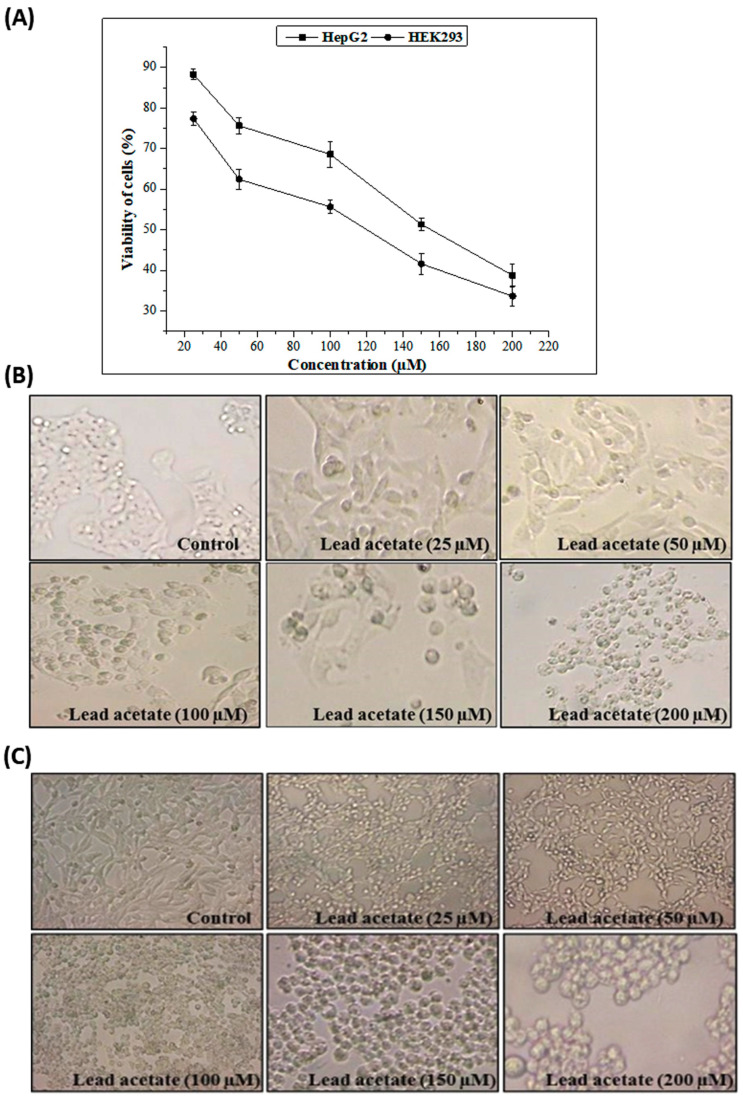
Cytotoxicity assay using HepG2 and HEK293 cells. (**A**) Cytotoxicity of different concentrations of lead acetate. Data are expressed as the mean ± standard deviation of triplicate measurements. (**B**) Morphologic changes in HepG2 cells after treatment with different concentrations of lead acetate. (**C**) Morphological changes in HEK293 cells after treatment with different concentrations of lead acetate.

The effects of different concentrations of lead acetate (LA) on HepG2 and HEK293 cell viability are shown in Figure 2. HepG2 and HEK293 cell viabilities were proven to significantly decrease (*p* < 0.05) with increasing concentrations of lead acetate in the growth medium from 25 to 200 µM. This metal, at 25 to 200 µM, killed 88–38% of the HepG2 and 77–33% of the HEK293 cells after 24 h of incubation.

### 2.4. Cytoprotective Effect of the Extract against Apoptotic Cell Death Due to Lead Acetate

**Figure 3:**
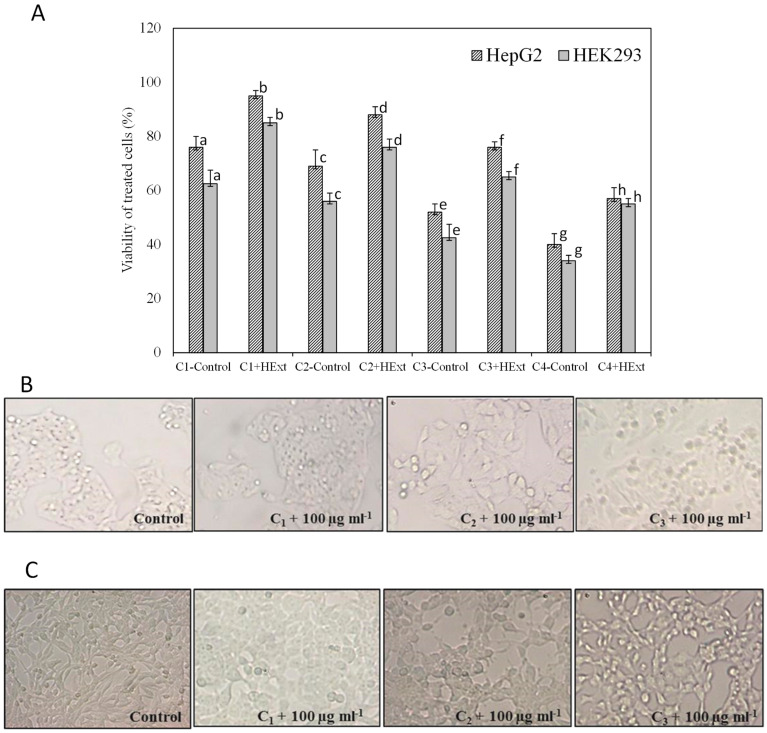
Protective effect of *Halamphora* extract (HExt) against lead-acetate-induced toxicity to HepG2 and HEK293 cells. (**A**) Viability of cells treated with different concentrations of lead acetate, 50 µM, 100 µM, 150 µM, and 200 µM (C1, C2, C3, and C4, respectively), and HExt (100 µg mL^−1^). (**B**) Morphological changes in HepG2 cells pretreated with HExt before the administration of different concentrations of lead. (**C**) Morphological changes in HEK293 cells pretreated with HExt before the administration of different concentrations of lead. All data are expressed as the mean ± SD. Different letters represent significant differences (*p* < 0.05) between each treatment and the control (cells treated with lead acetate).

The protective effect of HExt against lead-acetate-induced toxicity in HepG2 and HEK293 cells is shown in Figure 3. The HExt at 100 µg mL^−1^ significantly reduced (*p* < 0.05) the apoptotic cell death that is induced by this metal.

### 2.5. Effects of Extract Treatment on Lipid Peroxidation Levels in the Liver and Kidney

The MDA levels were significantly increased (*p* < 0.05) in the liver and kidney in LA-treated animals compared with the MDA levels in the control group (Table 2). This increase, after treatment with the extract of *Halamphora* sp., was significantly lowered (*p* < 0.01 and *p* < 0.05) by 20% and 21% in the liver and kidney, respectively.

### 2.6. Effects of Extract Treatment on Antioxidant Enzyme Activities in the Liver and Kidney

Lead intoxication in rats caused a significant decrease (*p* < 0.05) in SOD, CAT, and GPx activities in the liver and kidney compared to the control animals (Table 2). The extract of *Halamphora* sp. induced significant increases (*p* < 0.05) in the SOD activity of the liver and kidney by 40% and 71%, respectively, when compared to the control animals. Moreover, extract treatment also led to an increase in CAT activity by 96% and 48% and GPX activity by 41% and 43% in the rat livers and kidneys in Group IV (LA + HExt), respectively.

### 2.7. Effects of Extract Treatment on the Serum Biochemical Parameters in Rats

The ASAT, ALAT, ALP, and LDH activities in the serum of LA rats are shown in Table 3. They underwent significant increases of 147%, 119%, 106%, and 90%, respectively, when compared with the control rats (*p* < 0.01). The coadministration of *Halamphora* sp. extract and LA was found to bring about marked decreases in terms of the four indices of liver toxicity. Moreover, in serum, when compared with the control rats, the LA rats were noted to undergo significant increases (*p* < 0.01) of 53% and 101% in terms of the creatinine and urea levels, respectively. Interestingly, administering HExt simultaneously with LA reversed this increase, and the levels returned to normal. The findings from histological analysis further confirmed the positive effects of this supplement on the liver and kidney.

### 2.8. Histological Study

#### 2.8.1. Hepatocyte Histology

**Figure 4:**
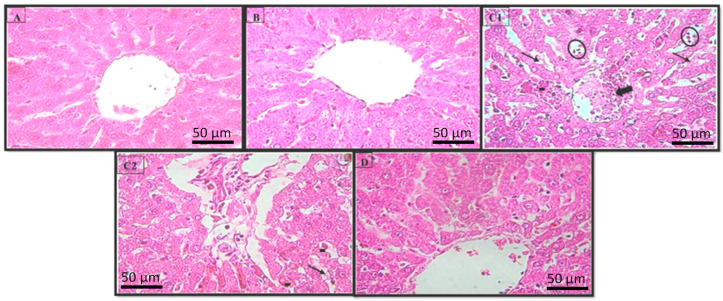
Histopathology of the rat liver (H & E × 400). Photomicrographs of the liver sections from (**A**) Group I, (**B**) Group II, (**C1**,**C2**) Group III, and (**D**) Group IV. Necrotic focus (circles), cytolysis (arrows), inflammatory cells (hyphen), and the congested central veins (large arrows). Optic microscopy: H & E (×400). H & E: hematoxylin and eosin.

The histological study revealed that the rat livers in the control group had normal hepatocytes (Figure 4A), with the presence of a portal tract, vein, and small artery. The sections prepared from the livers of rats treated with HExt alone showed a preservation of the normal structure of the liver (Figure 4B). The liver sections of the rats treated with 5 mg kg^−1^ of LA revealed cytolytic changes with the necrotic foci, inflammatory cells, and congested central veins (Figure 4C1,C2). The rats treated with LA + HExt showed a prominent improvement in hepatocyte histology, as is demonstrated by the necrotic areas markedly decreasing and the slight inflammatory cell infiltrations (Figure 4D).

#### 2.8.2. Kidney Effect

**Figure 5:**
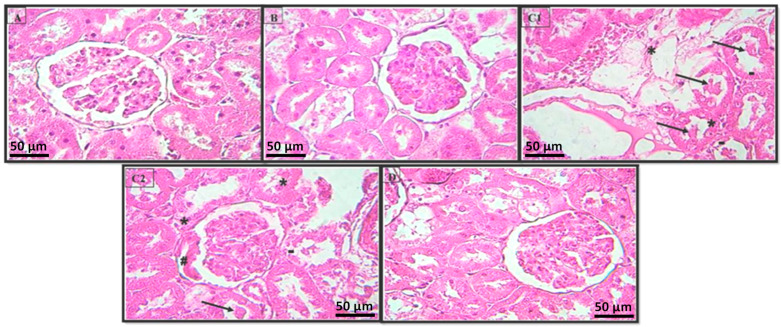
Histopathology of the rat kidney (H & E × 400). Photomicrographs of the kidney sections from (**A**) Group I, (**B**) Group II, (**C1**,**C2**) Group III, and (**D**) Group IV. Intraluminal cellular desquamation (arrows), a fibronecrotic small focus that has Bowman’s space (hashtag), and the vacuolar degeneration lesion of tubular cells (apoptosis) (hyphen) and tubular necrosis (stars). Optic microscopy: H & E (×400). H & E: hematoxylin and eosin.

The kidney sections of the control rats proved that the natural structures of the glomeruli and renal tubules were maintained (Figure 5A). The rats treated with the extract alone showed a normal histological pattern in the kidneys (Figure 5B). Intoxication with LA caused vacuolar degeneration lesions on the tubular cells (nephrosis) and intraluminal cellular desquamation (Figure 5C1); the presence of a small fibronecrotic focus that filled Bowman’s space (synechia) (Figure 5C2); and lesions that affected certain glomeruli. The coadministration of HExt and LA prevented these histopathological changes in the kidney tissues (Figure 5D).

## 3. Discussion

In this work, the phytochemical characterization of *Halamphora* sp. ethanol extract showed that it is rich in phenols and flavonoids (the 38.27 mg GAE g^−1^ extract and 17.69 mg QE g^−1^ extract, respectively). The total phenol content in the HExt was more significant than the phenolic contents in the ethanol extracts of certain Moroccan marine microalgae, which range from 8.2 to 32 mg GAE g^−1^ extracts [29]. The high levels of polyphenols and flavonoids in *Halamphora* sp. ethanol extract may be due to the high-salinity (100 PSU) culture conditions and the extraction conditions [1].

Moreover, the *Halamphora* sp. extract was found to be rich in chlorophylls, mainly chlorophyll (a) and carotenoids. Bacillariophyceae have exhibited high levels of carotenoids—namely, β-carotene and xanthophylls—thus indicating powerful antioxidant activity [30]. Boukhris et al. [1] demonstrated that *Halamphora* extract has strong antioxidant activity via four in vitro assays: DPPH and ABTS radical scavenging capacities, ferric-reducing antioxidant power, and β-carotene bleaching inhibition tests. Moreover, for *Halamphora* sp., 80% of the ethanolic extract exhibited high antiradical power with an IC50 value of 0.23 ± 0.07 mg mL^−1^ for the DPPH scavenging, and an IC50 of 2.61 ± 0.64 mg mL^−1^ for the ABTS radical scavenging.

The distribution pattern for the fatty acids in the *Halamphora* sp. extract reflected its richness in monounsaturated fatty acids (49.893%), followed by palmitoleic acid C16:1 (42.066%). The *Halamphora* sp. extract was also rich in saturated fatty acids (38.677%), followed by palmitic acid C16:0 (29.464%); in addition, the extract contained a significant percentage of polyunsaturated fatty acids (10.960%). High levels of palmitoleic acid and other bioactive fatty acids were also found in the fusiform morphotype of the Bacillariophyceae *Phaeodactylum tricornutum* [31]. The various saturated and unsaturated fatty acids present in numerous plant-derived food products have been evaluated for their antioxidant activities and they have shown substantial effects compared to the tested positive controls. Saturated fatty acids such as palmitic acid have shown high antioxidant activity [14].

In this study, the MTT test showed that HExt at <250 µg mL^−1^ did not impose a significant effect on the apoptotic death of HepG2 and HEK293 cells that were not treated with lead acetate. The results showed that HExt applied at 100 µg mL^−1^ protected HepG2 and HEK cells against Pb-induced damage. Thus, pretreating these cells with HExt followed by exposure to Pb protected the cells from the toxic effects of this metal, which suggests that this extract has the highest antiradical activity. The evaluation of the hepatonephroprotective activity of this species can be considered novel.

In the second part of the present work, we investigated the hepatonephroprotective effect of this extract against the toxic effects of lead exposure in vivo. The results showed that subchronic exposure to a low dose of lead acetate (5 mg kg^−1^ b.w. per day—(i.p)) for 3 weeks induced a significant increase in lipid peroxidation (MDA levels) in the liver and kidney. Previously, Omobowale et al. [19] indicated that the most important consequence of Pb-induced oxidative stress in the liver is lipid peroxidation. In addition, Pb exposure causes alterations in membrane integrity and fatty acid composition, and is associated with an increase in MDA levels in the liver and kidney [32,33,34]. Notably, Matović et al. [22] found that Pb causes lipid peroxidation, mainly via ROS generation such as H_2_O_2_ and OH• (although the experimental data also indicated the involvement of nitrogen species). In this study, the administration of *Halamphora* sp. extract at a dosage of 2 mg kg^−1^ b.w. per day for 3 weeks protected against lipid peroxidation in the liver and kidney. Moreover, the protective effect of HExt against the lipid peroxidation caused by lead toxicity may be attributed to its antioxidant activities due to its high palmitic acid content. In addition, olive oil is also rich in palmitic acid and has high antioxidant properties [35]. We assumed that the protective effect of fatty acids from HExt may be in maintaining and restoring the cells’ integrity.

Moreover, Pb exposure induced a decrease in GPx activity, which might have emanated from the Se-mediated detoxification of Pb when the Se level was insufficient to maintain optimal GPx activity [36,37]. The results showed that HExt plays an important role in regulating antioxidant capacity by changing the activities of SOD, CAT, and GPx. Such a regulatory ability was thoroughly evidenced in *Spirulina* by El-Tantawy [24]. The protective effect of HExt against lead toxicity may also be attributed to the chelating effect of this metal. Previously, it was suggested by Anantharaj et al. [38] that diatoms have a high capacity to absorb metals and can solve metal toxicity problems in aquatic ecosystems. Moreover, HExt is rich in unsaturated fatty acids that could potentially be employed as antioxidant agents [29]. In addition, Attia and Nasr found that fatty acids could maintain normal enzymatic activity levels, such as those of SOD and CAT [39]. The antioxidant effects of fatty acids by scavenging free radicals and inhibiting lipid peroxidation were previously reported by Pauwels and Kostkiewicz [40]. Since fatty acids play protective roles in the liver and kidney, they have been widely used in clinical preoperative and total parenteral nutrition [41,42].

Liver enzymes such as ALAT, ASAT, and ALP are markers of liver function and integrity [43,44]. In the present study, there was a significant increase (*p* < 0.01) in these enzyme levels in the lead-treated group (LA) compared to the control group. The elevated plasma levels of hepatic marker enzymes indicate cellular leakage and a loss in the functional integrity of the hepatic membrane architecture [44]. Damage to the hepatocyte membrane causes many of these enzymes to be released into circulation [43]. LDH, a marker of cell damage, is composed of subunits from red blood cells, the liver, the intestine, and the kidneys. Thus, the increase in LDH activity caused by Pb exposure was most likely a result of the breakdown of red blood cells [45]. ALP and transaminases are sensitive indicators of cellular injury and their elevation in serum leads to the consequent necrosis of hepatocytes occurring, thus leading to their leakage during toxin-induced insult to the liver [46]. In the present study, Pb treatment caused a highly significant increase in transaminases and ALP activity in serum, accompanied by a fall in their activities in liver tissue. Our results are in agreement with the above report. Hepatocellular degenerative changes, marked as necrotic foci, congested central veins, inflammatory cell infiltration, and cytolysis (Figure 4) observed in the histopathological evaluation in the present work are well corroborated by biochemical investigations.

Urea and creatinine levels were significantly increased (*p* < 0.01) in the lead-treated group (LA) compared to the control group. The increase in urea and creatinine contents due to lead acetate administration indicates that the kidney function was affected [33,47]. An increase in serum uric acid and creatinine concentrations in the blood may be due to a reduction in glomerular filtration in the kidney, and may also reflect dysfunction of the kidney tubules [5]. In this study, the elevation of creatinine levels in the blood of treated rats may be due to damage of the kidney cells and/or impaired kidney function under the toxic effect of lead acetate. Marked congestion and other degenerative changes were observed in the kidneys (Figure 5). The coadministration of HExt attenuated Pb-induced hepatonephrotoxicity—as shown via the elevations in the urea and creatinine levels and ASAT, ALAT, ALP, and LDH activities, which returned to near normal—in the Group IV rats. These results are in agreement with those found by El-Tantawy [24], who proved the beneficial role of *Spirulina* on enzymatic changes in rats.

In addition, cotreatment with HExt improved the histological alterations induced by lead, which could be attributed to the antioxidant and metal-chelating efficacy of this extract. The fatty acids EPA(n-3) and ArA(n-6) alleviate the symptoms of several inflammatory diseases [11]. The improvement in the histological picture of the groups of rats that were treated with *Halamphora* extract may be due to the fact that it contains many effective chemical compounds, especially phenolic compounds, carotenoids, and fatty acids (EPA-ArA), which are antioxidants that neutralize oxidative damage by scavenging free radicals and preventing fat peroxidation. Notably, Karadeniz et al. [48] reported that treatment with *Spirulina platensis* prevented the histopathological and enzymatic changes in the liver of rats that were harmed by cadmium.

In this paper, the diatom *Halamphora* sp. extract was clearly implicated in treating lead intoxication in liver and kidney cells. Therefore, this extract can be applied as a food or feed additive for outdoor aquacultures in an industrial region and can be recommended as a potential pharmaceutical additive product.

## 4. Materials and Methods

### 4.1. Chemicals

All chemicals used for the experiment were of analytical grade. Lead (in acetate form) was obtained from Standard Fine Chemicals, Bhoisar, Mumbai, India. The 5,5′-Dithiobis (2-nitrobenzoic acid) (DTNB), L-glutathione (reduced form), and all other chemicals were purchased from Sigma Chemical Co. (St. Louis, MO, USA).

### 4.2. Microalgal Isolation and Culture Conditions

Bacillariophyceae *Halamphora* sp. was isolated via micromanipulation and via serial dilution from pond C4-1 of the Sfax Solar Saltern, which has average salinity of 107 PSU. The Sfax Solar Saltern is located on the central eastern coast of Tunisia, approximately 34°39′ N and 10°42′ E. The ponds are shallow (20–70 cm deep) with salinity ranging from 37 to 400 g L^−1^ due to hypersaline conditions [49]. These ponds are connected by pipes and channels along a 12 km section of the sea cost.

The *Halamphora* was cultivated in batches in autoclaved artificial seawater, which was enriched with a F/2 nutrient medium, sodium silicate (Na_2_SiO_3_), and a trace metal solution [50]. Samples were cultured for 15 days at a salinity of 100 p.s.u. at 25 °C with a light/dark (L/D) cycle of 16 h:8 h, and were under cool white fluorescent light intensity of 60 µmols photons m^−2^ s^−1^. The *Halamphora* biomass was separated from the culture media via centrifugation (4500× *g*, 10 min); then, the pellet was washed twice with distilled water and centrifuged again at 4500× *g* for 10 min. The pellet was freeze-dried and stored at −70 °C.

### 4.3. Microalgal Extract, Preparation, and Characterization

#### 4.3.1. Extraction of Biomolecules

Extracts from the biomass samples were obtained via a solid–liquid extraction procedure inspired by the method of [51]. In the first step, both apolar and polar compounds were extracted using 80% ethanol. For this, 2 g of freeze-dried biomass was ground using a mortar and pestle and then extracted under agitation in the dark with 20 mL of ethanolic extract at 50 °C for 1 h. After centrifugation (4500× *g*, 10 min), the pellet was resuspended in 2 mL of the ethanol/water mixture and then extracted for a second time via maceration. The two extracts were pooled and stored at −20 °C prior to analysis and then concentrated under reduced pressure using a rotary evaporator to a dry condensed residue. The dried sample was weighed and stored at −20 °C prior to analysis.

#### 4.3.2. Determination of the Total Phenol Content in the Extract

The total phenol content in the HExt was determined using the Folin–Ciocalteu method [52]. Briefly, 0.2 mL of the extract was mixed with 1 mL of Folin–Ciocalteu reagent (diluted 1:10, *v*/*v*) followed by the addition of 0.8 mL of sodium carbonate (7.5%, *w*/*v*). After incubation in the dark, the absorbance was measured at 760 nm. The total phenolic content of the algae extract was expressed as the mg of gallic acid equivalents per g of dry extract (mg GAE g^−1^ extract); this was achieved using a calibration curve constructed with gallic acid. All samples were analyzed in triplicate.

#### 4.3.3. Determination of the Total Flavonoid Content in the Extract

The total flavonoid content was determined according to the modified method of Zhishen et al. [53]. Briefly, 0.4 mL of the extract was mixed with 120 µL of 5% sodium nitrite and 120 µL of 10% aluminum chloride, followed by the addition of 0.8 mL of 1 M sodium hydroxide. After incubating the reaction mixture at room temperature for 6 min, the absorbance was measured at 510 nm. The total flavonoid content in the extract was expressed in terms of catechin equivalents (mg g^−1^ of the dry extract). All samples were analyzed in triplicate.

#### 4.3.4. Determination of the Content of Pigments in the Extract: Chlorophyll (a) and Carotenoids

The pigments were determined according to Lichtenthaler [54]. After incubation of the methanolic extract (99.9%) in the dark for 24 h, the pigment content was determined by measuring the absorbance at 470, 652.4, and 665.2 nm, which were corrected for turbidity by subtracting the absorbance at 750 nm.

#### 4.3.5. Estimation of the Fatty Acid Profile in the Extract via GC–MS

The extract was dried under nitrogen flow. The dry extract was collected in heptane (10 mL), and after which, the fatty acid methyl ester (FAME) content was obtained by performing a basic transesterification protocol [1].

### 4.4. In Vitro Cytotoxicity Assay

#### 4.4.1. Cell Lines and Culture

Human hepatocellular carcinoma cells (HepG2) and human embryonic kidney cells (HEK293) were grown in 25-cm^2^ T-flasks (Falcon, Becton Dickinson, East Rutherford, NJ, USA) containing MEM and DMEM-F12, respectively, supplemented with 10% FCS, 1% L-glutamine, and 50 µg mL^−1^ gentamycin sulfate. The cells were incubated in a 37 °C CO_2_ incubator in an humidified atmosphere of 5% CO in 95% air.

#### 4.4.2. In Vitro Cytotoxicity Assay

After seeding, HepG2 and HEK293 cells were exposed to 50, 100, 150, 200, 300, and 400 µg mL^−1^ *Halamphora* extract. After 24 h of exposure, the 3-[4,5 dimethylthiazol-2-yl]-2,5-diphenyl tetrazolium bromide (MTT) test was used to determine the cell viability according to Carmichael et al. [55]. The reduction of MTT to a blue formazan product via mitochondrial dehydrogenase in viable cells was determined by measuring the absorbance at 560 nm at the indicated times after reagent addition. The cell viability in the treated samples was expressed as a percentage of the control (normal cells). Data represent the mean ± SD of at least three independently treated cell cultures.

#### 4.4.3. Cell Treatment

Cell suspensions were mixed with different concentrations of lead acetate in a multi-well plate. The final lead acetate concentrations ranged from 25 to 200 µM. Wells containing cells with lead acetate served as negative controls. For the correlated groups, the cells were pretreated with HExt at 100 µg mL^−1^ for 24 h, followed by treatment with different concentrations of lead acetate for 24 h in the presence of the extract. The cultures were incubated (5% CO, 37 °C) for 24 h before the determination of cell viability using the MTT cell proliferation assay. The optical density (OD) was measured at 570 nm using a Synergy™ HTX Multi-Mode microplate reader.

Cell proliferation was calculated according to the following formula:

$$Viability\left(\%\right)=\frac{Average\;of\;Test\;Sample\;\left(OD\right)-Average\;of\;Blank\;Sample\;(OD)}{Average\;of\;Control\;Sample\;\left(OD\right)-Average\;of\;Blank\;Sample\;(OD)}\times 100$$

### 4.5. Animals and In Vivo Treatment

Adult male Wistar albino rats weighing 210–265 g were obtained from the Central Pharmacy (SIPHAT, Tunisia). They were caged individually at 22 ± 3 °C with light/dark periods of 12 h and minimum relative humidity of 40%. The animals had free access to a commercial pellet diet (SICO, Tunisia) and water. The general guidelines for the use and care of living animals in scientific investigations were followed (Council of European Communities, 2010). The handling of the animals was approved by the Tunisian Ethical Committee for the Care and Use of Laboratory Animals.

#### 4.5.1. Experimental Design

A total of twenty-four male rats were allocated randomly into four experimental groups of six rats each.

Group I (Con): control animals given a standard diet and water;

Group II (HExt): animals receiving HExt via gastric gavage daily for 3 weeks at a dosage of 2 mg kg^−1^ body weight (b.w.);

Group III (LA): animals receiving lead acetate at a dosage of 5 mg kg^−1^ b.w. via daily intraperitoneal injection (i.p.) for 3 weeks;

Group IV (LA + HExt): animals receiving lead acetate at a dosage of 5 mg kg^−1^ b.w. (i.p.) and HExt at a dosage of 2 mg kg^−1^ b.w. via daily gastric gavage for 3 weeks.

At the end of the experimental period, all animals were anesthetized and then sacrificed via decapitation to minimize handling stress. The trunk blood was collected. The serum was separated via centrifugation (1500× *g*, 15 min, 4 °C), and then frozen and stored at −20 °C until use for biochemical assays with a physiological saline solution. 

A portion of each organ was homogenized (10% *w*/*v*) using an Ultra Turrax homogenizer in 0.01 M ice-cold sodium potassium phosphate buffer (pH 7.4). Homogenates were centrifuged at 10,000× *g* for 20 min at 4 °C. The resulting supernatants were aliquoted and stored at −80 °C for biochemical analysis.

#### 4.5.2. Determination of Stress Biomarkers

Malondialdehyde (MDA) concentrations were determined spectrophotometrically according to Draper and Hadley [56]. The protein content in the liver and kidney was determined according to the method of Lowry et al. [57], using bovine serum albumin as a standard.

#### 4.5.3. Antioxidant Enzyme Status

Superoxide dismutase activity was assayed, according to Beyer and Fridovich [58], by measuring its ability to inhibit the photoreduction of nitroblue tetrazolium (NBT). In this assay, one unit of SOD was defined as the amount required to inhibit the photoreduction of NBT by 50%. Riboflavin (0.26 mM final concentration) was added to start the reaction, and the absorbance at 560 nm was recorded for 20 min. The activity was expressed as U SOD/mg protein at 25 °C.

Catalase activity was measured according to Abei [59]. A total of 20 µL of liver or kidney homogenate (approximately 1.5 mg of protein) was added to 1 mL of phosphate buffer (0.1 M, pH 7) that contained 100 mM H_2_O_2_. The rate of H_2_O_2_ decomposition was followed by measuring the decrease in absorbance at 240 nm for 1 min. The enzyme activity was calculated using an extinction coefficient of 0.043 mM^−1^ cm^−1^, and was expressed in international units (I.Us.), i.e., in the µmoles of H_2_O_2_ that were destroyed/min/mg protein at 25 °C.

The glutathione peroxidase activity was measured via the procedure of [60]. Each reaction mixture (1 mL) containing 0.3 mL of phosphate buffer (0.1 M, pH 7.4), 0.2 mL of 2 mM glutathione, 0.1 mL of sodium azide (10 mM), 0.1 mL of H_2_O_2_ (1 mM), and 0.3 mL of liver or kidney homogenate was prepared. After incubation at 37 °C for 15 min, the reaction was terminated by adding 0.5 mL of 5% TCA. Tubes were centrifuged at 1500× *g* for 10 min, and the supernatant was collected. A total of 0.1 mL of reaction supernatant, 0.2 mL of (0.1 M, pH 7.4), and 0.7 mL of DTNB (0.4 mg mL^−1^) were added. After mixing, the absorbance at 420 nm was recorded; furthermore, the enzyme activity was expressed as the nmoles of the GSH min^−1^ mg^−1^ protein.

#### 4.5.4. Biochemical Markers in Serum

The serum levels of aspartate aminotransferase (ASAT), alanine aminotransferase (ALAT) [31], creatinine, and urea and the activities of alkaline phosphatase (ALP) [61] and lactate dehydrogenase (LDH) were measured in frozen aliquots of serum via standardized enzymatic procedures using commercial kits from Biomaghreb, Ariana, Tunisia (refs. 20043, 20047, and 20012, respectively) via an automatic biochemistry analyzer (Vitalab Flexor E, Vital Scientific, Spankeren, The Netherlands).

#### 4.5.5. Histological Analysis

Liver and kidney tissues were fixed in a 10% formaldehyde solution immediately after being removed. The washed tissues were dehydrated in an increasing gradient of ethanol and then cleared in toluene. The tissues were then embedded in molten paraffin wax. Sections were cut to a thickness of 3 mm and stained with hematoxylin and eosin.

### 4.6. Statistical Analysis

Statistical analyses were performed using SPSS software (version 20). Data are expressed as the mean ± standard deviation, and were analyzed via one-way ANOVA. Differences were considered to be significant at *p* < 0.05.

## 5. Conclusions

*Halamphora* sp. extract is rich in polyphenols, flavonoids, chlorophyll (a), carotenoids, and saturated and unsaturated fatty acids. It can be considered to be a powerful source of antioxidants both in vitro and in vivo. The present research work has shown that HExt, at a dose of 100 µg mL^−1^, has a protective effect against lead-induced oxidative stress and toxicity in human liver and kidney cells. Extracts from *Halamphora* sp. can be used as potent natural antioxidants and can protect against lead toxicity in the liver and kidney. The extract is rich in bioactive compounds and is safe for application in vivo; these factors make it advantageous to use HExt to add value to food, feed, the pharmaceutical industry, and biomedicine, and for the treatment of lead intoxication.

## Figures and Tables

**Table 1:** Phytochemical characterization of diatom *Halamphora* extract.

Biochemical Components	*Halamphora* Extract
Yield (%DW)	18.20 ± 0.21
**Phenolic compounds**	
Polyphenols (mg GAE g^−1^ extract)	38.27 ± 2.21
Flavonoids (mg QE g^−1^ extract)	17.69 ± 0.70
**Pigments**	
Carotenoids (mg g^−1^ extract)	2.19 ± 0.05
Chlorophyll (a) (mg g^−1^ extract)	6.11 ± 0.10
**Fatty acids**	
C14:0	0.175 ± 0.4
C15:0	0.851 ± 0.3
C16:0	29.464 ± 0.3
C17:0	1.655 ± 0.5
C18:0	2.317 ± 0.3
C20:0	0.463 ± 0.2
C24:0	3.752 ± 0.2
**Total SFA**	**38.677 ± 0.6**
C14:1(n-5)	1.072 ± 0.2
C16:1(n-7)	42.066 ± 0.7
C17:1(n-8)	0.446 ± 0.1
C18:1(n-9)	4.993 ± 0.4
C19:1(n-9)	1.316 ± 0.2
**Total MUFA**	**49.893 ± 1.3**
C16:2(n-7)	1.459 ± 0.3
C18:2(n-6)	0.567 ± 0.1
C20:4(n-6) (ArA)	2.867 ± 0.2
C20:5(n-3) (EPA)	6.067 ± 0.3
**Total PUFA**	**10.960 ± 0.5**

SFAs: saturated fatty acids, MUFAs: monounsaturated fatty acids, PUFAs: polyunsaturated fatty acids. EPA: eicosapentaenoic acid, DW: dry weight, and ArA: arachidonic acid. Data are expressed as the mean ± the standard deviation of triplicates.

**Table 2:** The oxidative stress and antioxidant enzyme status of *Halamphora* extract in rat livers and kidneys.

Parameters and Treatments	MDA (nmol of MDA mg^−1^ Protein)	SOD (U SOD mg^−1^ Protein)	CAT (µmoles H_2_O_2_ Destroyed min^−1^ mg^−1^ Protein)	GPx (nmoles of GSH min^−1^ mg^−1^ Protein)
**Liver**				
**Group I**	1.014 ± 0.10	64.736 ± 5.08	272.156 ± 39.59	0.027 ± 0.001
**Group II**	1.064 ± 0.09	65.145 ± 4.47	269.520 ± 42.98	0.027 ± 0.002
**Group III**	1.397 ± 0.05 #	45.305 ± 4.14 #	132.054 ± 30.97 #	0.020 ± 0.001 ##
**Group IV**	1.110 ± 0.02 **	66.323 ± 2.98 *	259.020 ± 18.75 **	0.028 ± 0.002 *
**Kidney**				
**Group I**	0.960 ± 0.05	42.897 ± 2.23	89.344 ± 10.27	0.022 ± 0.001
**Group II**	0.902 ± 0.07	41.857 ± 4.70	90.501 ± 9.37	0.020 ± 0.001
**Group III**	1.233 ± 0.07 #	24.613 ± 4.41 #	61.078 ± 4.09 #	0.016 ± 0.0005 ###
**Group IV**	1.057 ± 0.07 *	50.946 ± 4.00 *	98.424 ± 8.10 **	0.023 ± 0.0004 ***

Values are given as the mean ± SD for each group of six animals. Group III compared to Group I: # *p* < 0.05; ## *p* < 0.01; and ### *p* < 0.001. Group IV compared to Group III: * *p* < 0.05; ** *p* < 0.01; and ***p < 0.001.

**Table 3:** The serum biochemical parameters of the control (Group I) and treated groups of rats.

Groups	Group I	Group II	Group III	Group IV
ASAT (IU/L)	79 ± 12.8	80.2 ± 14.6	195.8 ± 38.9 ##	123.2 ± 9.8 *
ALAT(IU/L)	14.8 ± 2.3	19.7 ± 2.7	32.6 ± 5.3 ##	22.2 ± 1.4 *
ALP (U/L)	103 ± 22.1	117.5 ± 19.7	212.8 ± 16.1 ##	132 ± 26.6 *
LDH (U/L)	780.7± 114.2	802.2 ± 133.8	1489.8 ± 168.8 ##	1004.3 ± 75.4 *
Urea (mmol/L)	3.0 ± 0.44	4.1 ± 0.23	6.1 ± 0.58 ##	4.23 ± 0.38 *
Creatinine (µmol/L)	20 ± 1.9	19.3 ± 1.8	30.6 ± 2.97 ##	22.8 ± 2.5 *

Results are expressed as mean ± SD. Number of determinations (n = 6). No significant difference between Group II and Group I. Significant differences between other groups are mentioned as follows: Group III compared to Group I: ## *p* < 0.01. Group IV compared to Group III: * *p* < 0.05.

## Data Availability

Data is contained within the article.
